# Early detection of cervical cancer in western Kenya: determinants of healthcare providers performing a gynaecological examination for abnormal vaginal discharge or bleeding

**DOI:** 10.1186/s12875-021-01395-y

**Published:** 2021-03-11

**Authors:** Emily Mwaliko, Guido Van Hal, Hilde Bastiaens, Stefan Van Dongen, Peter Gichangi, Barasa Otsyula, Violet Naanyu, Marleen Temmerman

**Affiliations:** 1 Department of Reproductive Health, School of Medicine, Moi University, Box 4606, Eldoret, 30100 Kenya; 2 Epidemiology and Social Medicine, Social Epidemiology and Health Policy, University of Antwerp, Universiteitsplein 1, 2610 Wilrijk, Antwerp, Belgium; 3 Department of Primary and Interdisciplinary Care, Faculty of Medicine and Health Sciences, University of Antwerp, Gouverneur Kinsbergen Centrum, Doornstraat 331 - 2610 Wilrijk, Antwerp, Belgium; 4 Department of Biology, Evolutionary Ecology Group, University of Antwerp, Universiteitsplein 1, 2610 Wilrijk, Belgium; 5 DVC Academic Research & Extension, Technical University of Mombasa, Mumbasa, Kenya; 6 Ghent University, Ghent, Belgium; 7 Department of Surgery, School of Medicine, Moi University, P.O. Box 4606, Eldoret, 30100 Kenya; 8 Department of Sociology Psychology and Anthropology, School of Arts and Social Sciences, Moi University, P.O. Box 3900, Eldoret, 30100 Kenya; 9 Department of Public Health and Primary Care, Ghent University, Ghent, Belgium; 10 Department of Obstetrics and Gynaecology, Aga Khan University, P O. Box 00100, Nairobi, Kenya

**Keywords:** Theory of planned behaviour, Health care providers, Cervical cancer, Early detection, Health care seeking delays, Primary health care, Abnormal uterine bleeding, Kenya

## Abstract

**Background:**

In western Kenya, women often present with late-stage cervical cancer despite prior contact with the health care system. The aim of this study was to predict primary health care providers’ behaviour in examining women who present with abnormal discharge or bleeding.

**Methods:**

This was a cross-sectional survey using the theory of planned behaviour (TPB). A sample of primary health care practitioners in western Kenya completed a 59-item questionnaire. Structural equation modelling was used to identify the determinants of providers’ intention to perform a gynaecological examination. Bivariate analysis was conducted to investigate the relationship between the external variables and intention.

**Results:**

Direct measures of subjective norms (DMSN), direct measures of perceived behavioural control (DMPBC), and indirect measures of attitude predicted the intention to examine patients. Negative attitudes toward examining women had a suppressor effect on the prediction of health workers’ intentions. However, the predictors of intention with the highest coefficients were the external variables being a nurse (β = 0.32) as opposed to a clinical officer and workload of attending less than 50 patients per day (β = 0.56).

In bivariate analysis with intention to perform a gynaecological examination, there was no evidence that working experience, being female, having a lower workload, or being a private practitioner were associated with a higher intention to conduct vaginal examinations. Clinical officers and nurses were equally likely to examine women.

**Conclusions:**

The TPB is a suitable theoretical basis to predict the intention to perform a gynaecological examination. Overall, the model predicted 47% of the variation in health care providers’ intention to examine women who present with recurrent vaginal bleeding or discharge. Direct subjective norms (health provider’s conformity with what their colleagues do or expect them to do), PBC (providers need to feel competent and confident in performing examinations in women), and negative attitudes toward conducting vaginal examination accounted for the most variance. External variables in this study also contributed to the overall variance. As the model in this study could not explain 53% of the variance**,** investigating other external variables that influence the intention to examine women should be undertaken.

**Supplementary Information:**

The online version contains supplementary material available at 10.1186/s12875-021-01395-y.

## Background

Kenya has a high incidence and mortality from cancer of the cervix. In 2018, GLOBOCAN estimated that 5250 (19.7%) of 26,688 new cases of cancer in women were cervical cancer. Cervical cancer is the leading cause of female cancer mortality, accounting for 17.5% (3286/18,772) of all cancer deaths among women in Kenya [1]. Cervical cancer is preventable through screening and vaccination. However, vaccination programs have not taken off nationwide in Kenya [2]. Screening uptake is low (14%) and is lacking in rural areas, and many women present with cervical cancer at an advanced stage [3–5].

It is estimated that about 95% of women in developing countries have never had a screening test. In addition, 80% of women with newly diagnosed cancer in developing countries already have advanced disease. Research among patients with cancer has shown that even when women present with genital tract symptoms like bleeding or discharge, no examination has been done and many have been treated repeatedly without a concrete diagnosis [6–9].

Numerous factors can influence the implementation of evidence-based guidelines in clinical practice among health care professionals. This includes their knowledge, training, individual motivational predispositions, remuneration, and workplace organizational contexts. It is important to assess the practices of primary health care providers as they are the health professionals that women contact first in rural areas [10–14].

Medical training dictates that when a patient is bleeding, the health practitioner should examine the patient to find from what or where the bleeding is coming. However, women in sub-Saharan Africa who have cervical cancer can bleed for months without undergoing a vaginal examination while being attended by a health provider [15]. Provider delay has been studied less than patient delay. As Unger-Saldana et al. revealed [16], the affected individuals tend to be blamed for their health problems and a lack of medical attention. This begs the questions why this situation exists, how can it be improved, how can the threshold of suspected serious disease be raised when a woman presents with abnormal vaginal bleeding, and how can we promote more frequent gynaecological examinations by health care professionals [17].

To answer these questions, further theoretically based research is needed, to better inform the design of interventions aimed at changing the behaviour of health care professionals. As many clinical practice decisions are individual professional decisions, it would be useful to obtain a better understanding of the individual mechanisms involved in the adoption of new behaviours, using social psychology theories.

The theory of planned behaviour (TPB) [18] was chosen for this study because it is focused on motivation. The TPB proposes that motivation determines behaviour, and therefore, the best predictors of behaviour are factors that predict or determine motivation. The TPB has been used in other clinical domains to explain individuals’ behaviour and factors that can be changed; however, to date, there are no studies regarding clinician’s behaviour in gynaecological practice [18–24].

Therefore, we conducted the present study, using the TPB to determine those factors that influence primary health care practitioners’ intention to examine women with recurrent vaginal bleeding or discharge when they present for medical consultation, and to identify the beliefs associated with this intention.

## Methods

This was a quantitative, cross-sectional, questionnaire-based study conducted in private and public health facilities in western Kenya. The study site, Bungoma East sub-county, is a typical rural area in Kenya as far as the hurdles and challenges in the health system. Most people in this sub-county are farmers.

The study population comprised all nurses and clinical officers working in Bungoma East County in private clinics, dispensaries, health centres, and faith-based hospitals. These health care providers offer antenatal care, maternity care, family planning, and general outpatient care. Nurses and clinical officers are the main staff in these facilities.

Clinical officers undergo a 4-year basic training course in clinical medicine at the diploma level whereas registered nurses complete 3.5 years of training; the enrolled community nurses had completed 2 years of training. All these professional groups have received training in midwifery, which was part of the inclusion criteria if working in the county.

Qualified nurses and clinical officers working within the county in clinical areas (emergency/family planning/trauma) were included in the present study. We excluded those involved solely in administrative work.

### Study sample

The target sample size was based on Green’s [25] recommendation. That author proposed the following rule: *N* ≥ 50 + 8 m for multiple correlation and *N* ≥ 104 + m for partial correlation, where m refers to the number of predictor variables in the model. This means that, because we were predicting intention (to perform a gynaecological examination when a woman consults with abnormal vaginal discharge or bleeding) using the three predictors [attitude (toward performing a gynaecological examination), subjective norms (whether there is social pressure to perform an examination) and perceived behavioural control (whether the provider feels confident in performing a gynaecological examination)] of the TPB, we would need 50 + 24 = 74 providers in the sample for a robust multiple R, and 104 + 3 = 107 for the significance test relating to individual beta weights for the predictors.

To recruit the study participants, a sampling frame was constructed using the official list of the cadres working in each health facility. Table 1 shows the number of health care providers targeted.

**Table 1:** Sampling frame of health care facilities and providers

Health facilities (Bungoma East)	No. of health facilities in this study (Norms & Standardsfor level of facility)	No. of health care providers to beinterviewed	Total
Dispensaries (13/15)	13 (2 registered nurses)	2 each	26
Health centres (5/5)	5 (2 clinical officers, 14 registered nurses)	9 (2 clinical officers, 7 registered nurses) each	45
Hospitals (2)	1 (2 clinical officers, 8 registered nurses)	6 (2 clinical officers, 4 registered nurses)	6
Private medical clinics (9/10)	9 (by owner(s), either clinical officer or registered nurse)	1 (either clinical officer or registered nurse)	9
**Total: 29** (3 non-operational)			**86**

Random sampling was used, with the aim of interviewing nurses mainly in the emergency/family planning/trauma areas. There were very few clinical officers and private health care providers (i.e., private clinic owners) and we aimed to interview them all, as they were available. One day prior to visiting each facility, an appointment was booked by phone with the person in charge of the facility. We met with that individual as well as other nurses depending on the duty roster or work schedule, in consideration of the work shifts of providers and staff shortages. We actively sought to interview nurses and clinical officers.

#### Study variables

**Fig. 1:**
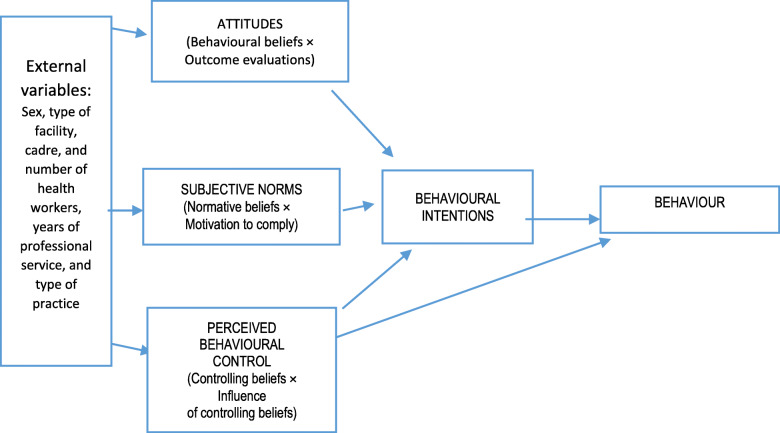
Theory of Planned Behaviour (Ajzen, [27]). Legend: Three variables (attitudes; subjective norms; and perceived behavioural control) which the theory suggests will predict the intention to perform a behaviour. Intentions are the precursors of behaviour. External variables - these might influence intention (from literature). The theory of planned behaviour applies to behaviour that one strives to perform. The intention to achieve a goal (in this case to perform a gynecological examination) is not the same as to actually perform it. Attitude is one of the three variables that the theory suggests predicts intention. Attitude towards the act (performance) is the precursor (mental event) of intention. Intention is the pathway attitudes take towards actually performing the act/behaviour (observable action). But intention is not action. Intention is what immediately precedes the behavior: someone has the intention to perform a specific behaviour, and shortly thereafter, the behaviour is performed, unless some unexpected situations occur. Attitude is more distal and a more ‘global’ concept. In the Theory of Planned Behaviour, the expectation of what a certain behaviour will result in and the value attributed to that expected result, determine the attitude. E.g.: when a gynaecologist expects that performing a medical exam in a woman with vaginal complaints will help this woman, the gynaecologist has a positive expectation of performing that exam. When the gynaecologist expects that doing a medical exam will make no difference, or even harm the woman, he/she has a negative expectation of performing that exam. When the gynaecologist also values a favourable outcome for the woman as important, he/she has a positive value. A positive expectation combined with a positive value, will lead to a positive attitude, which in turn, will lead to a bigger chance that he/she will have the intention to perform a medical exam

We used the manual - ‘*Constructing questionnaires based on the theory of planned behaviour: A manual for health services researchers’ -* in the development of the questionnaires [26, 27]. All three constructs of the TPB in Fig. 1 were used to elicit the salient beliefs of each respondent. Additional file 1 shows the questions analysed when constructing the questionnaire. A questionnaire was developed to identify the determinants for performing a gynaecological examination. The questionnaire was checked for internal consistency on the direct measures. The Cronbach alpha was 0.67.

There is no perfect relationship between intention and the actual performance of a behaviour. Intention, which has measurable variables, is used as the proxy measure of behaviour. The model by Ajzen provides a way to predict behaviour using three variables, even though actual behaviour is not readily measurable. These three variables (attitudes, subjective norms and perceived behavioural control) are psychological constructs. Attitudes indicate beliefs about the consequences of performing a behaviour. Subjective norms are an individual’s estimate of the social pressure to perform or not perform the behaviour. Perceived behavioural control is about the individual’s confidence in performing the behaviour. In this study, the behaviour of interest is performing a gynaecological examination when a woman consults with abnormal vaginal discharge or bleeding.

The number of questions developed for each construct and examples to each are shown in Additional file 2. The questions were rated on a seven-point Likert scale and negatively worded responses were recoded so that higher scores were inclined towards performing the behaviour. Intention simulation questions were ten clinical scenarios that described patients presenting with abnormal vaginal bleeding or discharge. The respondents were to decide whether they would or would not do a gynaecological examination. The responses were summed to create a total score.

This questionnaire was piloted among health care providers at Moi Teaching and Referral Hospital, to check for clarity and comprehension and Bungoma East county health providers. The final questionnaire – Additional file 3, was then administered to the study participants.

### Data collection

During data collection, a trained research assistant distributed the questionnaires to participants and checked for completeness of the returned surveys. Several visits were made to reach as many providers as possible. Despite these efforts, several questionnaires were not filled, for several reasons: the providers were busy with scheduled clinical duties, on leave, in workshops or conducting outreach; or the questionnaire was too long. No questionnaires were posted or left for the provider to complete on their own in their free time as previous experience has shown that respondents are unlikely to complete the surveys in such cases [28, 29].

### Data management and analysis

To investigate the relationships among the study variables, structural equation modelling (SEM) was applied using the lava package in R. First, a measurement error model was constructed using factor analyses. This allowed construction of the hypothesized latent variables and examination of how the observed variables reliably reflected them. Second, these latent constructs, as well as sex (male vs. female), profession (clinical officer vs. nurse), length of qualification (< 5 years, > 5 years), cadre of colleagues (nurses only, clinical officers only, both nurses and clinical officers), number of nurses (< 4, 4 or more), number of clinical officers (none, 1–2, > 2), number of patients per day (< 50, > 50) and type of facility (health centre, dispensary, private clinic) were used in a structural equation model to explain the variation in the intention to examine women (expressed as a proportion of intention on the basis of the 10 scenarios). Estimates were obtained using maximum likelihood, and all variables were standardized such that estimates of effect sizes were obtained on a comparable scale.

To determine the specific beliefs with the greatest influence on intentions, the intention variable was dichotomized using a median split. The median of the intention variable was 6, so we created two groups of equal sample size of health care providers with either low intention (a score of 5 or less) or high intention (a score of 6 or higher). We then univariately compared the health care provider characteristics (experience, sex, profession, type of services, workload) and the intention to examine women (low vs. high intention group).

## Results

Out of the health facilities recorded at the district office 3 were non-operational leaving 29 on record as operating. In the sampling frame only one hospital was sampled (the other is a teaching hospital and therefore not included). The health facilities were therefore 28. While collecting data one health centre was not operational yet and one private clinic was closed. There were 18 public facilities and 8 were private. There were 13 dispensaries, 4 health centres and 1 mission hospital. Two additional facilities were non-functional.

**Fig. 2:**
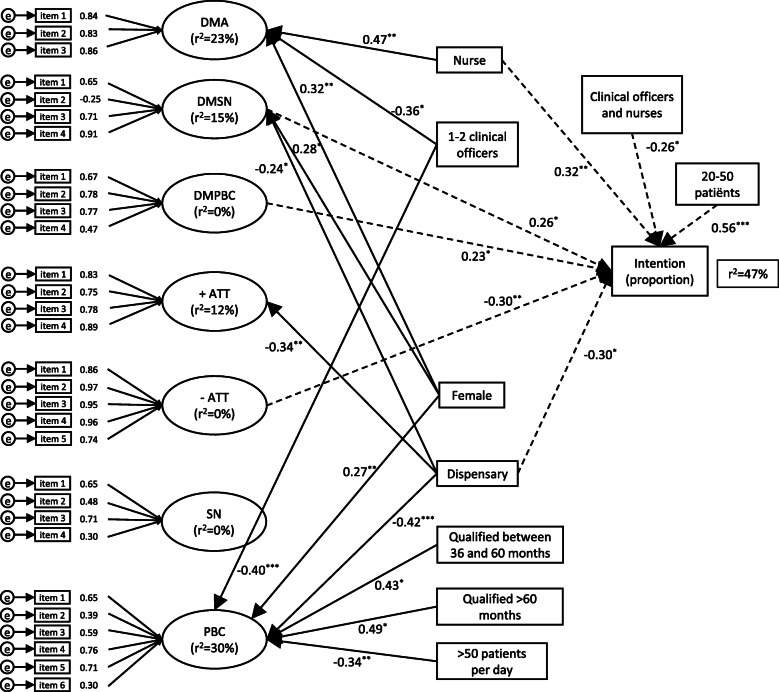
Graphic representation of the SEM exploring the relationships among the predictor variables of the TPB and intention to do a gynaecological examination. Legend: Ovals indicate latent constructs; rectangles indicate observed constructs. Error values associated with each are indicated as small circles with the letter “e” inside them. Standard coefficients are shown above the paths between constructs showing positive and negative associations. Factor loadings are indicated between latent constructs and the indicators. The variation explained in the model by the six latent constructs, r^2^, is provided. The direction of the coefficients – positive or negative, show whether the relationship between the predictor and outcome is positive or negative and the number indicates the degree to which the predictor affects the outcome. Bold arrows show variation in latent variables whereas dashed arrows show variation in the intention to perform a gynaecological examination. Standardized effect sizes and their statistical significance (* *p* < 0.05, ** *p* < 0.01, *** *p* < 0.001) are shown. DMA, direct measures of attitude; DMSN, direct measures of subjective norm; DMPBC, direct measure of perceived behavioural control; +ATT, positive measures of attitude (indirect); −ATT, negative measures of attitude (indirect); SN, indirect measures of subjective norm; PBC, indirect measures of perceived behavioural control

The results of the structural equation model for the determinants predicting the intention to perform gynaecological examinations can be found in Fig. 2.

As Fig. 2 shows, 47% of the variation in the intention to perform a gynaecological examination was explained by the model (r^2^). The measurement model on the basis of the factor analyses showed generally high factor loadings. Nevertheless, some items had low loadings in comparison with others (the acceptable loading being 0.3), as below:

- Item 2 in DMSN (I feel social pressure to perform a vaginal examination in a patient who presents with abnormal vaginal bleeding/discharge.) (− 0.25).
- Item 4 in direct measures of perceived behavioural control (DMPBC) (Whether I do a vaginal examination or not is entirely up to me.) (0.47).
- Items 2 and 4 in indirect measures of subjective norms (Patients with recurrent abnormal vaginal bleeding would disapprove or approve of my doing a vaginal examination (0.48). Other Clinical Officers and Nurses do not do or do vaginal examinations in patients who consult with vaginal discharge/bleeding.) (0.30).
- Items 2 and 6 in indirect measures of perceived behavioural control (A lack of skills/knowledge/practice is unlikely/likely to influence whether I perform a vaginal examination (0.39). A lack of skills/practice/knowledge makes it much more difficult/much easier to perform a vaginal examination.) (0.30).

For four of the latent constructs, variation was explained by the other explanatory variables (Fig. 2). The direct measures of attitude (DMA) score were significantly higher in nurses (β = 0.47) than in clinical officers and higher in female (β = 0.32) than male health professionals; this score was lower when one or two clinical officers were present as compared with none or more than two clinical officers (β = − 0.36) in the health facility. These explanatory variables explained 23% of the variation in DMA scores.

Positive attitudes scores were negatively predicted in scores from dispensaries (β = − 0.34) than in health centres and private practices, explaining 12% of the variation in positive attitudes towards examining women. DMSN prediction scores were higher in female (β = 0.28) than in male health providers and suppressing in effect from dispensaries (β = − 0.24) than in health centres and private practices. These explained 15% of the variation in prediction of subjective norms.

Perceived behavioural control prediction scores were lower in dispensaries (β = − 0.42) than in health centres and private practices, when 1 or 2 clinical officers were present (β = − 0.40) as compared with none or more than 2 clinical officers, and when more than 50 patients (β = − 0.34) were treated per day than a lower number of patients. In addition, perceived behavioural control was higher in female (β = 0.27) than in male health providers and with length of qualification 36–60 months and >  60 months, explaining 30% of the variation.

The variation in intention to examine women was explained by the following seven variables:

- Higher with < 50 patients (β = 0.56) treated per day than > 50 patients per day and higher among nurses scores (β = 0.32) than in clinical officers
- Positively related to DMSN scores (β = 0.26) and DMPBC scores (β = 0.23)
- Negatively predicted by scores from dispensaries (β = − 0.30) than in health centres and private practices and when both clinical officers and nurses formed the cadre (β = − 0.26)
- Negatively predicted by negative attitudes (β = − 0.30) scores (a more negative attitude resulted in a lower intention to examine women).

Table 2 shows the baseline characteristics of health care providers. There was a total of 10 clinical officers and 56 nurses. Nearly 73% of health care providers were female and most had more than 5 years’ working experience. Fourteen percent reported working in a facility that attended fewer than 20 patients per day.

**Table 2:** Baseline characteristics of health care providers

Characteristic	Frequency	%
Cadre		
Clinical officer	10	15.2
Nurse	56	84.8
Sex		
Male	18	27.3
Female	48	72.7
Length of work experience after qualification (mo.)		
< 60	19	28.8
≥ 60	47	71.2
Cadre working in the facility		
Nurses only	29	43.9
Clinical officer only	0	0.0
Both nurses and clinical officers	37	56.1
No. of nurse colleagues^a^		
≤ 3	26	39.4
4 +	37	56.1
No. of clinical officer colleagues^a^		
None	29	43.9
1–2	28	42.4
3 +	5	7.6
Nature of health facility		
Public	57	86.4
Private	9	13.6
No. of patients per day^a^		
< 20	9	13.6
20–50	25	37.9
> 50	31	47.0

^a^Missing cases

On the basis of the univariate analysis (Table 3) we found no difference in the proportions of experienced vs. inexperienced health care providers, men vs. women, profession, type of services offered, or workload between the groups of high and low intention. Furthermore, clinical officers and nurses were equally likely to examine women. There was no statistical evidence demonstrating a difference in the proportion of male participants in public and private facilities (29.8% vs. 11.1%, *p* = 0.425).

**Table 3:** Univariate tests of associations between characteristics of health care providers and intention to conduct a gynaecological examination (low vs. high intention groups on the basis of the median level of intention). The null hypothesis tested is that the percentage of health care providers in both groups does not differ between the different level of the tested characteristics. The percentages in the columns of the two groups of level of intention are calculated relative to the total number of health care providers in each row (and thus add up to 100% for each row in the table). The percentages in the column with the total numbers are relative to the total number of health care providers

Variable	Level of intention			
	Low (≤ 5)	High (> 6)	Total	*p*-value
	*N* = 33	*N* = 33	*N* = 66	
Experience (y)				
< 5	24 (51.1%)	23 (48.9%)	47 (71.2%)	0.786
≥ 5	9 (47.4%)	10 (52.6%)	19 (28.8%)	
Sex				
Male	8 (44.4%)	10 (55.6%)	18 (27.3%)	0.580
Female	25 (52.1%)	23 (47.9%)	48 (72.7%)	
Professional				
Clinical officer	5 (50.0%)	5 (50.0%)	10 (15.2%)	> 0.999
Nurse	28 (50.0%)	28 (50.0%)	56 (84.8%)	
Type of services				
Private	2 (22.2%)	7 (77.8%)	9 (13.6%)	0.073
Public	31 (54.4%)	26 (45.6%)	57 (86.4%)	
Workload (number of patients/day)^a^				
≤ 50	20 (58.8%)	14 (41.2%)	34 (52.3%)	0.105
> 50	12 (38.7%)	19 (61.3%)	31 (47.7%)	

^a^Missing value

## Discussion

The TPB was the theoretical foundation for this study. We used the key concepts of Ajzen: attitudes, subjective norms, perceived behavioural control, and intention [18]. In this study, we sought to identify the motivational factors associated with the intention of primary care providers to perform a gynaecological examination when a woman presents with recurrent abnormal vaginal bleeding (i.e., consultation for the same complaint more than once).

Standardized regression weights of the TPB constructs indicated that direct measures of subjective norms were the best predictor of intention, followed by direct measures of perceived behavioural control. We can postulate that if these two are improved and there is a change in the negative attitudes associated with performing a gynaecological examination, then the intention to examine women will improve. However, these are hypotheses and a causal relationship cannot be extrapolated from this path analysis; studies are needed regarding the impact of these variables on actual behaviour. The TPB constructs and other variables in the model explained 47% of the variance in intention; 53% of this variance cannot be explained by this model. Other studies using this theory to examine health provider’s intentions in clinical contexts have reported an explained variance of between 19 and 81% (frequency-weighted mean) [30, 31]. In this study, the TPB constructs were not the best predictors of health providers’ intentions to conduct vaginal examinations. The intention to examine in this study is associated with external factors, both indirect (female sex, type of facility, type of cadre) and direct (workload, mixed cadre, and being a nurse).

Behavioural intention should result in the behaviour of conducting gynaecological examinations; however, behaviour was not evaluated in this study. As in the study by Godin et al., factors other than the TPB constructs may influence the decisions of health providers. Habit (whether to act out a behaviour) has been shown to influence behaviour performance. In this study, the habit was failure to conduct gynaecological examinations despite symptoms or clinical indications [30, 32].

Indirect measures of subjective norms probably assessed insufficient or inappropriate beliefs as this did not predict intention and had no direct or indirect effects (R^2^ = 0%).

The path model also shows several factors with low loadings. These were the opinions of colleagues regarding what they think should be done and what they actually do, as well as social pressure to conduct gynaecological examinations. As suggested in other studies, this may be because health care providers may make decisions without being influenced, even by practice guidelines. Scores for confidence and other factors that determine whether an examination is carried out may have been low because of a lack of resources or a proper environment in which to do a gynaecological examination (a lack of instruments or private rooms) as well as the age/sex of the provider relative to that of the patient [30, 31]. The reason for these low scores could also be owing to the way these questions were scored, although standard scoring procedures were followed [26].

Bleeding may be a symptom of reproductive tract pathology, including cervical cancer. It is recommended that before a diagnosis of abnormal uterine bleeding is made using the PALM-COEIN classification (classifies causes of abnormal bleeding into structural and functional: Polyps, Adenomyosis, Leiomyoma, Malignancy and hyperplasia, Coagulopathy, Ovulatory dysfunction, Endometrial, Iatrogenic, and Not yet classified), lesions of the cervix must be ruled out. The PALM-COEIN classification helps in investigations and selecting treatment modalities. Therefore, even examining patients under age 25 years (recommended age of screening initiation) also helps to establish the diagnosis [33, 34]. By the time a woman is diagnosed with stage 3B cervical cancer, there is spread from the cervix to the vaginal walls. If a health provider had performed a vaginal examination much earlier, an earlier diagnosis could likely have been made.

In rural areas, most women initially visit dispensaries and health centres manned by nurses and clinical officers [15, 35, 36]. We hypothesized that sex, the number of years of work experience, profession, workload (number of patients seen per day), and type of facility are factors that predict health care professionals’ intention to examine women. These are factors external to the TPB, and we predicted the value of these factors [31].

According to the study findings, being a nurse and a workload of 20–50 patients per day was associated with more frequent gynaecological examinations conducted in women (the recommended workload, according to the norms and standards of health service delivery of the Ministry of Health Kenya, is 17 patients per day) [37]. There is a shortage of health care providers, especially in rural facilities. Most health centres and dispensaries are run by female workers. In our study, there were more female than male providers, but we reasoned that positive attitudes and the motivation to examine women would be stronger in female providers who attend a woman that is bleeding. Reluctance to be seen by male providers, either by the woman herself or her partner, has been reported in other studies [38–40].

Negative attitudes toward vaginal examination, as seen in the indirect measures of attitude, were associated with being a dispensary as opposed to health centre and having a mixed cadre of both nurses and clinical officers. These negative attitudes had a suppressor effect on the predicted variance, i.e., these contributed negatively to the intention to perform examinations in women. Factors such as patient preference and resource constraints also influence prediction in the TPB [20, 30].

There were direct effects on perceived behavioural control from the external variables that were hypothesized to predict intention. Having worked for more than 5 years and being female had a positive influence on the performance of vaginal examinations. However, being a dispensary and seeing more than 50 patients per day suppressed the variance in perceived control and confidence in performing examinations [41, 42]. More than 36 countries in sub-Saharan Africa have been classified by the World Health Organization as having a critical shortage of health workers. This includes Kenya, which is a constraint in implementing public health interventions, especially in primary care.

How can we improve gynaecological examination of women and detection of abnormalities, to refer women with cervical cancer earlier? The first problem to address is the staff shortages in rural areas. Increasing the staff would reduce the workload, thereby creating more time for thorough history taking and examination of women.

Women will still need screening and early detection, even once a human papillomavirus vaccination program is in place. However, establishing such a program will take time and those who are sexually active now must be examined if they present with recurrent bleeding and discharge. Therefore, health providers should be encouraged to do away with any negative attitudes and to develop the motivation, competence, and confidence to perform gynaecological examinations when necessary [39, 40].

Working in a dispensary was found to be associated with negative attitudes toward examining patients. Motivation to do what other colleagues expect, and even the capability and confidence to examine patients, is reduced in the presence of negative attitudes. This may not be owing to the provider’s attitudes alone but may also be influenced by the lack of supplies and equipment. Availability of the proper environment may prompt health workers to make the effort to examine patients. Therefore, another area to address is improving the supply/reserves of dispensaries, which are relatively smaller than health centres but are easier for the population to reach [42].

Indirect measures of attitude included questions on the negative outcomes of performing a vaginal examination. This must be addressed via knowledge provided to health care professionals. For example, fear of spreading infection or causing further bleeding is not a valid reason to avoid conducting a vaginal examination. This is also clinically related behaviour, whose performance may depend on several factors, such as those highlighted by Godin et al. [30, 31]. Past behaviour was not a variable in those studies, which would have indicated whether a habit is common among practitioners and whether providers have actually been examining patients during a longer period.

### Study strengths and limitations

One main strength of this study is that we created our questionnaire using a guide developed based on the well-established theory of planned behaviour (TPB). For the first time, we developed and used case scenarios in this study; additional studies are needed to clarify whether these can be considered adequate as a proxy to measure intention.

Path analysis examines the contribution of specific variables within a specific model, without testing for causality (a single regression will not test the contribution of that path to the model. One model cannot be compared with another based on the R^2^). However, path analysis fits with the findings of other studies using the TPB to predict health providers’ intentions.

This was a cross-sectional study and therefore has limitations inherent to this study design. Although we used the TPB to guide and support the correlations and associations found in our study, we do not provide evidence regarding the cause of health care providers not performing a gynaecological examination. Indeed, the main limitation in the study is the sample size. The study was underpowered which is a limitation on the results. However, we used a validated instrument and the statistical conclusions we made reflect this. The associations we found in this study need to be verified using a larger sample (for more robust results) within the same population where the content of the beliefs was drawn from.

Participants completed the questionnaires during regular working hours and their answers may be unreliable or may have been different if given more time. Providers who had busy schedules or were unavailable were not included in the analyses. These limitations, together with the small sample size, may have impacted the results.

Although limited by these factors, this study demonstrates the potential of the TPB to identify the predictors of health providers’ intention to perform gynaecological examinations in women. This study provides direction for further research, which should be carried out using an adequate random sample, to better understand the behaviours of primary health care providers regarding gynaecological examinations.

### Importance of the findings for public health

The behaviour of primary health care workers in performing gynaecological examination among patients with symptoms of abnormal bleeding or discharge may lead to earlier diagnosis in cervical cancer and other genital tract diseases. Our study findings will inform policy makers of interventions to improve clinical effectiveness through identifying modifiable factors like knowledge, attitudes, self-efficacy, and a lack of resources, which can be used to eventually improve the intention-to-action of performing gynaecological examinations.

In this study, subjective norms were associated with intention, which suggests that providers felt that examining patients is expected and colleagues also perform gynaecological examinations of patients. Perceived control also predicted intention; however, several barriers were found. Eliminating these barriers and supporting feelings of confidence in conducting vaginal examinations should be included in the interventions. Initially, this will be based on guidelines, but eventually such behaviours should become habitual.

## Conclusions

In predicting the intention to examine women who present with abnormal vaginal bleeding or discharge, the TPB appears to be a suitable theoretical basis for investigating this behaviour. Our study findings indicated that DMSN, DMPBC, and indirect measures of attitude could only explain 47% of the variance in the intention to perform a gynaecological examination when a woman consults for recurrent abnormal vaginal bleeding. This variance was also explained by several external variables: the number of patients attended per day, being a nurse, being a dispensary facility, and cadres with both nurses and clinical officers working together. Resource constraints (as evidenced by workload and type of facility, i.e., dispensary) within the health facilities had a negative association with intention.

No other studies have explicitly used the TPB to investigate health providers’ behaviours regarding gynaecological examination of women. Therefore, our study serves as an important baseline for other research involving clinical procedures in reproductive health. Our findings also provide research-based evidence in how TPB constructs can be exploited to best improve patient care.

## Supplementary Information

**Additional file 1.** Constructing the questionnaire using the manual based on the TPB. Open-ended questionnaire for generating questions on salient beliefs of the study population and case scenarios using intention simulation method as measure of actual behaviour.

**Additional file 2.** Number and examples of the questions developed to measure each construct. Number and examples of the developed questions and the measuring scale used for direct and indirect measures of attitudes, subjective norms, and perceived behavioural control in the questionnaire.

**Additional file 3.** Study questionnaire. This is the final questionnaire used to collect data on the direct and indirect measures of the constructs of the TPB and the external variables. The questions were mixed and some reverse-coded.

## Data Availability

The datasets used and/or analysed during the study are available from the corresponding author on reasonable request.

## Abbreviations

WHO: World Health Organization
PALM-COEIN: Polyps, Adenomyosis, Leiomyoma, Malignancy and hyperplasia, Coagulopathy, Ovulatory dysfunction, Endometrial, Iatrogenic, and Not classified yet
TPB: Theory of Planned Behaviour
DMA: Direct measures of attitude
DMSN: Direct measures of subjective norms
DMPBC: Direct measures of perceived behavioural control
IMA: Indirect measures of attitude
IMSN: Indirect measures of subjective norms
IMPBC: Indirect measures of perceived behavioural control
RN: Registered Nurse (Kenya Registered Community Health Nurse): Acquired a diploma in nursing and community health and can undergo further training to upgrade to specialist courses e.g., midwifery, anaesthesia etc.
CO: Clinical Officer: - A cadre of professionals that undertook 4 years in a medical college and acquired a Diploma in Clinical Medicine and Surgery and Community Health and undergo a one-year internship in a teaching hospital. They work under medical officers (Doctors) and can undergo courses to upgrade to specialists e.g., anaesthesia, ophthalmology etc.
