# Supplementary material for: Early detection of cervical cancer in western Kenya: determinants of healthcare providers performing a gynaecological examination for abnormal vaginal discharge or bleeding
Source: BMC Fam Pract. 2021 Mar 11;22:52. doi: 10.1186/s12875-021-01395-y (PMC7953728; doi:10.1186/s12875-021-01395-y)
Supplement: Supplementary file 1 — Additional file 1. Constructing the questionnaire using the manual based on the TPB. Open-ended questionnaire for generating questions on salient beliefs of the study population and case scenarios using intention simulation method as measure of actual behaviour. [file 12875_2021_1395_MOESM1_ESM.docx]

**Additional file 1:**

Title: Constructing the questionnaire using the manual based on the TPB.

Description: Open-ended questionnaire for generating questions on salient beliefs of the study population and case scenarios using intention simulation method as measure of actual behaviour.

TACT principle: Doing a gynaecological examination when a woman consults complaining of recurrent/abnormal vaginal bleeding**.**

A: Attitudes

Direct measures of attitude

1. Conducting a vaginal examination in a patient with abnormal vaginal bleeding/discharge is

Harmful: 1. . 2. . 3. . 4. . 5. .6. . 7: beneficial

Good: 1. . 2. . 3. . 4. . 5. .6. .7 : bad (practice)

Pleasant: 1. . 2. . 3. . 4. . 5. . 6. .7 : unpleasant

Worthless : 1. . 2. . 3. . 4. . 5. . 6. . 7 : useful

Indirect measures of attitude (developed after elicitation study)

1. What do you believe are the advantages of performing a vaginal examination in a woman with recurrent abnormal vaginal bleeding/discharge?
2. What do you believe are the disadvantages of doing a vaginal examination?
3. Is there anything else you associate with your own views about performing a vaginal examination?

B: Subjective norms

Direct measures of subjective norms

A patient presents with recurrent abnormal vaginal bleeding/discharge:

1. Most people important to me think--

I should: 1. . 2. . 3. . 4. . 5. . 6. . 7 : should not

--do a vaginal examination.

1. It is expected of me that I do a vaginal examination when a patient has recurrent abnormal vaginal bleeding/discharge.

Strongly disagree: 1. . 2. . 3. . 4. . 5. . 6. .7 : strongly agree

1. I feel under social pressure to do a vaginal examination in a patient who presents with abnormal vaginal bleeding/discharge.

Strongly disagree: 1. . 2. . 3. . 4. . 5. .6. .7 : strongly agree

1. People who are important to me expect me to do a vaginal examination on a patient with recurrent vaginal bleeding or discharge.

Strongly disagree: 1. . 2. . 3. . 4. . 5. .6. .7 : strongly agree

Indirect measures of subjective norms (developed after elicitation study):

1. Are there any individuals or groups who would approve of your doing a vaginal examination in a patient with recurrent abnormal vaginal bleeding?
2. Are there any individuals or groups who would disapprove of your doing a vaginal examination in a woman with recurrent abnormal vaginal bleeding?
3. Is there anything else you associate with other peoples’ views about doing a vaginal examination in a patient with recurrent abnormal vaginal bleeding?

C: Perceived behavioural control

Direct measures of perceived behavioural control

Self-efficacy:

1. I am confident I could do a vaginal examination for a patient if I wanted to.

Strongly disagree: 1. . 2. . 3. . 4. . 5. .6. .7 : strongly agree

1. For me, performing a vaginal examination is

Easy: 1. . 2. . 3. . 4. . 5. .6. .7 difficult

Controllability:

1. The decision to do a vaginal examination is beyond my control.

Strongly disagree: 1. . 2. . 3. . 4. . 5. .6. .7 : strongly agree

1. Whether I perform a vaginal examination is entirely up to me.

Strongly disagree: 1. . 2. . 3. . 4. . 5. .6. .7 : strongly agree

Indirect measurement of perceived behavioural control (developed after elicitation study)

In a patient with recurrent abnormal vaginal bleeding:

1. What factors or circumstances would enable you to do a vaginal examination?
2. What factors or circumstances would make it difficult or impossible for you to do a vaginal examination?
3. Are there other issues that come to mind when you think about performing a vaginal examination?

D. Measuring Intention

Case scenarios (proxy) for measuring actual behaviour of health providers, using the intention simulation method

1. A 14-year-old girl comes to the health centre complaining of vaginal bleeding and abdominal pains for a week. She is accompanied by her mother who reports that she lives with her aunt during the week, as her school is nearer there. Menarche was at age 12 years.

Decision: Would you perform a pelvic examination?

| Yes |  |
| --- | --- |
| No |  |

2. A 65-year-old woman comes in complaining of vaginal spotting after sexual intercourse. Her spouse died 10 years ago but she has had a new partner the last 5 years. She has used K-Y jelly but the spotting recurs. After an online search, she wants to know if she can use a hormonal preparation. You have some free samples in the office and she has no other complaints.

Decision: Would you do a pelvic exam?

| Yes |  |
| --- | --- |
| No |  |

3. The first patient of the morning comes in complaining of lower back pain for 4 months. She is 39 years old and is on Depo-Provera for contraception. Her menses are usually irregular. She has been treated repeatedly with antibiotics for a blood-tinged discharge. She finished the last dose 6 weeks ago and she feels that she needs a new prescription.

Decision: Would you perform a pelvic examination?

| Yes |  |
| --- | --- |
| No |  |

4. Mrs. X comes in for a consultation complaining of a swollen left leg and pain on walking. She is 42 years old and has four children; her last delivery was six years ago. She has never married. Her method of contraception is combined pills, which she started 5 months ago because of bleeding from implants. Initially, she thought the pills were helping but of late, Sylate was added to control the irregular bleeding. She also complains of occasional constipation and back pains. Is a pelvic examination necessary?

| Yes |  |
| --- | --- |
| No |  |

5. Mrs. Z has heard about the cervical cancer vaccine and wants to know how and where to get it. She is 30 years old, para 3–0, and had a coil inserted 3 years ago. She has occasional blood-tinged discharge, which she was told was because of the coil. This has become worse and she is becoming scared about cancer as her grandmother died of breast cancer.

Decision: Would you do a pelvic examination?

| Yes |  |
| --- | --- |
| No |  |

6. Sara is a neighbour, aged 32 years with three children. She is married and uses condoms for contraception because she was told her blood pressure was high during her last pregnancy.

She has confided in you repeatedly about her irregular bleeding. She has come to report that the medications you gave her 2 months ago worked; now she thinks she is pregnant but she is not ready for another pregnancy. She has lower abdominal pains.

Is a pelvic examination necessary?

| Yes |  |
| --- | --- |
| No |  |

7. Mary arrives at the clinic complaining of lower abdominal pain and bleeding from the vagina. Her last normal menses were 3 years ago. She has had the pain for many months and she associates it with her workload. The bleeding is intermittent, but the last episode was 5 months ago; it lasted 2 weeks and left her weak. She feels much better and came in for her monthly prescription of hematinics and analgesics.

Would you conduct a check-up, including a pelvic exam?

| Yes |  |
| --- | --- |
| No |  |

8. Zippy was last seen at the clinic 5 years ago for infertility but was told her cervix was normal. She is 38 years old and her only child is 23 years old. She has missed her menses the last 2 months. She believes that she is pregnant and might lose the baby because she keeps spotting on and off.

Decision: Would you do a pelvic examination?

| Yes |  |
| --- | --- |
| No |  |

9. Jennifer has come in several times complaining of a burning sensation on passing urine. She has been treated repeatedly for infections. The last time, she was treated together with her spouse. Her partner refuses to use a condom. She says she has come in for her usual medications as the blood-stained discharge has recurred. She does not want to queue. She always takes the same drugs and says she is “cured”.

Can she do without a pelvic exam?

| Yes |  |
| --- | --- |
| No |  |

10. Sixty-five-year-old Ms. Nancy noticed a mass in her lower abdomen 1 year ago. She also had lower abdominal pains, which have become worse. She has been using traditional herbs, which stopped the bleeding. During her last visit, she was given something for the pain, which is bearable now. She has been referred because she has difficulty controlling her urine and her urinalysis revealed blood in the urine.

An ultrasound in more important; can a pelvic examination be deferred?

| Yes |  |
| --- | --- |
| No |  |
