# Supplementary material for: Early detection of cervical cancer in western Kenya: determinants of healthcare providers performing a gynaecological examination for abnormal vaginal discharge or bleeding
Source: BMC Fam Pract. 2021 Mar 11;22:52. doi: 10.1186/s12875-021-01395-y (PMC7953728; doi:10.1186/s12875-021-01395-y)
Supplement: Supplementary file 2 — Additional file 2. Number and examples of the questions developed to measure each construct. Number and examples of the developed questions and the measuring scale used for direct and indirect measures of attitudes, subjective norms, and perceived behavioural control in the questionnaire. [file 12875_2021_1395_MOESM2_ESM.docx]

**Additional file 2:**

**Title: Number and examples of the questions developed to measure each construct.**

| **Behaviour under study**: Performing a vaginal examination in a patient with recurrent/abnormal discharge or bleeding | | |
| --- | --- | --- |
| Constructs (number of questions) | | Example questions |
| Attitudes  Direct (4)  Scale: 1 to 7; harmful/beneficial; pleasant/unpleasant | Evaluative adjectives with a single stem that defines the behaviour (performing a gynaecological examination). | Performing a vaginal examination is harmful/beneficial. |
| Indirect (18)  Scale: 1 (unlikely) to 7(likely) and  -3 extremely undesirable to +3 extremely desirable | Strength of behavioural beliefs multiplied by outcome evaluations. The score is the mean of the sum of these. | If I do a vaginal examination, I will identify the source of bleeding.  If I do a vaginal examination, I can introduce/spread infection. |
| Subjective norms  Direct (4)  Scale: 1 to 7 | The opinions of important people about gynaecological examinations. The mean of the scores give the subjective norms score. | Most people important to me think I should perform vaginal examinations. |
| Indirect (8)  Normative beliefs: -  Scale: -3 (should not) to +3 (should)  Motivation to comply: -  Scale: 1 (not at all) to 7 (very much) | Individual/reference groups likely to apply pressure to perform gynaecological examinations.  Social pressure (what others think should be done and what they actually do) is multiplied by strength of the motivation to comply and the products summed to obtain an overall score. | Colleagues think I should perform a vaginal examination when a patient presents with recurrent bleeding.  Other clinical officers and nurses do not conduct gynaecological examinations. |
| Perceived control  Direct (4)  Scale: 1 to 7 | Confidence and ability to perform a gynaecological examination. Mean of the total score is the score for perceived control over behaviour. | I am confident that I can perform a vaginal examination if I wish.  The decision to do a gynaecological examination is beyond my control. |
| Indirect (14)  Control beliefs: - Scale: 1 (unlikely) to 7 (likely)  Perceived power: - Scale: -3 (less likely) to +3 (more likely) | Beliefs that make it difficult to perform gynaecological examinations. Each control belief is multiplied by the control factors and the products summed to give an overall score. | The unavailability of instruments makes it impossible to examine a patient.  When it is unclear how to manage a patient after the findings of a gynaecological exam, doing this exam becomes less likely. |
| Behavioural intention (10)  Scale: Yes/No | Case scenarios | Mary arrives at the clinic complaining of lower abdominal pain and bleeding from the vagina. Her last normal menses was 3 years ago. She has had pain for many months, and she associates it with her workload. The bleeding is intermittent but the last episode lasted for 2 weeks. She feels much better and came in for her monthly prescription of haematinics and analgesics.  Conduct a check-up, including a pelvic exam? |
