# Supplementary material for: Early detection of cervical cancer in western Kenya: determinants of healthcare providers performing a gynaecological examination for abnormal vaginal discharge or bleeding
Source: BMC Fam Pract. 2021 Mar 11;22:52. doi: 10.1186/s12875-021-01395-y (PMC7953728; doi:10.1186/s12875-021-01395-y)
Supplement: Supplementary file 3 — Additional file 3. Study questionnaire. This is the final questionnaire used to collect data on the direct and indirect measures of the constructs of the TPB and the external variables. The questions were mixed and some reverse-coded. [file 12875_2021_1395_MOESM3_ESM.docx]

Additional file 3:

**QUESTIONNAIRE FOR HEALTH PROVIDERS TPB STUDY**

Principle investigator: Dr Emily Mwaliko

Research assistant:

Date: Number:

Background information (external variables)

- Gender: …
- How long have you been qualified? …Months
- Are you a Clinical officer…Nurse…?
- Type of health facility (dispensary, health centre, private etc.) …
- How many nurses …. and clinical officers …work in this facility?
- How many outpatients per day (both new and reviews) do you see when on duty…?

**All questions refer to doing a vaginal examination (VE) in a patient with recurrent vaginal bleeding or discharge (irregular vaginal bleeding).**

INSTRUCTIONS:

e.g. Harmful 1: 2: 3: 4: 5: 6: 7: beneficial- This is how you rate your feelings concerning the statement along the range from harmful to beneficial. In the options -3..-2.…+2..+3 this is to what extent/how much you agree with the statement. There are no correct or wrong answers.

Please don’t circle two numbers together or the gap in between.

(Recurrent=irregular=abnormal. in this study)

1.Doing a VE in a patient with abnormal vaginal bleeding/discharge is

Harmful: 1. . 2. . 3. . 4. . 5. . 6. .7 : beneficial

Good: 1. . 2. . 3. . 4. . 5. . 6. .7 : bad (practice)

Worthless: 1. . 2. . 3. . 4. . 5. . 6. .7 : useful (procedure)

Pleasant: 1. . 2. . 3. . 4. . 5. . 6. .7 : unpleasant (for me)

**A patient presents with recurrent (abnormal) vaginal bleeding or discharge:**

1. The community’s beliefs/culture/age difference/sex difference

Should not: -3. .-2. . -1. . +1. . +2. . +3 : should

hinder a h/provider from doing a VE (in a patient with recurrent vaginal bleeding or discharge).

1. Unavailability of instruments/space/equipment makes it impossible to review a patient (with recurrent vaginal bleeding or discharge)

Unlikely: 1. . 2. . 3. . 4. . 5. . 6. .7 : likely

1. Lack of guidelines on management of recurrent vaginal discharge/bleeding deters one from doing a VE

Unlikely: 1. . 2. . 3. . 4. . 5. 6. .7 : likely

1. Age/sex difference between h/provider and patient is likely to make doing a VE

Less likely: -3. .-2. . -1. . +1. . +2. . +3 : more likely

1. When patients are uncooperative or there’s lack of consent, I am

Less likely: -3. .-2. . -1. . +1. . +2. . +3 : more likely to do a VE (in patients with recurrent vaginal bleeding or discharge)

1. Lack of Skills/practice/knowledge in doing a VE makes it

Much more difficult: -3. .-2. . -1. . +1. . +2. . +3 : easier to feel like doing it.

1. Most people important to me think--

I should: 1. . 2. . 3. . 4. . 5. . 6. .7 : should not

--do a vaginal examination.

1. Patients poor hygiene or other diseases makes it difficult to do a VE

Unlikely: 1. . 2. . 3. . 4. . 5. .6. . 7 : likely

1. (For me) Reassuring a woman that she is ‘okay’ after doing a VE (when they come for checkup (i.e., they have no complains) is

Extremely undesirable: -3. .-2. . -1. . +1. . +2. . +3: extremely desirable.

1. Knowing which important investigations to do is

Extremely undesirable: -3. .-2. . -1. . +1. . +2. . +3 : extremely desirable.

1. Introducing or spreading infection am doing a VE (in a patient with recurrent vaginal bleeding or discharge) is for me

Extremely undesirable: -3. .-2. . -1. . +1. . +2. . +3 : extremely desirable.

1. My colleagues approval of what I practice is important to me

Not at all: 1. . 2. . 3. . 4. . 5. . 6. .7 . : Very much

1. When there is a lack of equipment/space/instruments, I am

Less likely: -3. .-2. . -1. . +1. . +2. . +3 : more likely

to think of doing a VE (in a patient with recurrent vaginal bleeding or discharge)

1. When it’s unclear how to manage the patient after the findings of a VE (in a patient with recurrent vaginal bleeding or discharge), one is

Less likely: -3. .-2. . -1. . +1. . +2. . +3 : more likely…….: to do it.

1. The age/sex difference and cultural taboos between provider and patient is a reason to defer doing a VE (in a patient with recurrent vaginal bleeding or discharge)

Unlikely: 1. . 2. . 3. . 4. . 5. .6. .7 : likely

1. Colleagues (COs, Nurses) think I

Should not: -3. .-2. . -1. . +1. . +2. . +3 : should

do a VE when a patient has recurrent vaginal bleeding/discharge.

1. For male health providers, doing a procedure they are uncomfortable with is

Extremely undesirable: -3. .-2. . -1. . +1. . +2. . +3 : extremely desirable

1. When I have the history of presenting illness and previous treatments am certainly

Less likely: -3. .-2. . -1. . +1. . +2. . +3 : more likely

to do a VE in a patient with abnormal vaginal bleeding.

1. I am confident I could do a VE on patients if I wanted to;

Strongly agree: 1. . 2. . 3. . 4. . 5. . 6. .7 : strongly disagree

1. Patients poor hygiene or other suspected diseases makes doing a VE

Less likely: -3. .-2. . -1. . +1. . +2. . +3 : more likely

when they present with abnormal vaginal bleeding/discharge.

1. The community’s approval of what I practice as a health provider matters

Not at all: 1. . 2. . 3. . 4. . 5. . 6. .7: very much

1. I feel good when I reassure a woman that she is ‘okay’ after doing a VE for check-up (i.e., when they have no complains).

Unlikely: 1. . 2. . 3. . 4. . 5. . 6. .7: likely

1. Detecting the problem and referring the patient timely is:

Extremely undesirable: -3. .-2. . -1. . +1. . +2. . +3: extremely desirable

1. Patients with recurrent (abnormal) vaginal bleeding or discharge would

Disapprove: -3. .-2. . -1. . +1. . +2. . +3: approve

of my doing a vaginal examination.

1. Lack of consent/cooperation from a patient is a good reason not to do an VE (patient with recurrent vaginal bleeding or discharge)

Unlikely: 1. . 2. . 3. . 4. . 5. . 6. .7 : likely

1. If I do a VE (in a patient with recurrent vaginal bleeding or discharge) I can worsen the situation (more bleeding/discharge)

Unlikely: 1. . 2. . 3. . 4. . 5. . 6. .7: likely

1. Knowing the origin/source of bleeding is when a patient has abnormal vaginal bleeding is:

Extremely undesirable: -3. .-2. . -1. . +1. . +2. . +3: extremely desirable

1. Whether I do a VE or not is entirely up to me.

Strongly disagree: 1. . 2. . 3. . 4. . 5. . 6. .7: strongly agree

1. It is expected of me that I do a vaginal examination when a patient has recurrent abnormal vaginal bleeding/discharge.

Strongly disagree: 1. . 2. . 3. . 4. . 5. . 6. .7.: strongly agree

1. What patients think I should do for them matters to me

Not at all: 1. . 2. . 3. . 4. . 5. . 6. .7: very much

1. Worsening the bleeding/discharge during a vaginal examination (in a patient with recurrent vaginal bleeding or discharge) is

Extremely undesirable: -3. .-2. . -1. . +1. . +2. . +3: extremely desirable

1. Prescribing the right treatment/management for the patient is

Extremely undesirable: -3. .-2. . -1. . +1. . +2. . +3: extremely desirable

1. I feel under social pressure to do a vaginal examination in a patient who presents with abnormal vaginal bleeding/discharge.

Strongly disagree: 1. . 2. . 3. . 4. . 5. . 6. .7.: strongly agree

1. If I do a VE I can detect problems and refer the patient to the next level timely/sooner.

Unlikely: 1. . 2. . 3. . 4. . 5. . 6. .7.: likely

1. People who are important to me want me to do a vaginal examination on a patient with recurrent vaginal bleeding or discharge.

Strongly disagree: 1. . 2. . 3. . 4. . 5. . 6. .7: strongly agree

1. If I do a VE I can prescribe the right treatment/management

Unlikely: 1. . 2. . 3. . 4. . 5. . 6. .7: likely

1. Doing what other colleagues do is important to me

Not at all: 1. . 2. . 3. . 4. . 5. . 6. .7: Very much

1. Other Clinical Officers and Nurses

Do not do: -3. .-2. . -1. . +1. . +2. . +3: do

vaginal examinations in patients with abnormal vaginal bleeding or discharge.

1. For me to do a VE is

Difficult: 1. . 2. . 3. . 4. . 5. . 6. .7.: easy

1. Vaginal examination is an uncomfortable procedure for male health providers

Unlikely: 1. . 2. . 3. . 4. . 5. . 6. .7: likely

1. If I do a VE it will guide with important investigations

unlikely: 1. . 2. . 3. . 4. . 5. . 6. .7.: likely

1. The decision to do a VE is beyond my control

Strongly disagree: 1. . 2. . 3. . 4. . 5. . 6. .7: strongly agree

1. If I do a VE (in a patient with recurrent vaginal bleeding or discharge) I can introduce/spread infection:

Unlikely: 1. . 2. . 3. . 4. . 5. . 6. .7: likely

1. Doing a VE (in a patient with recurrent vaginal bleeding or discharge) causes excessive pain and trauma to the patient

Unlikely: 1. . 2. . 3. . 4. . 5. . 6. .7: likely

1. Lack of skills/knowledge/practice in doing a VE does not inspire one to examine a patient (with recurrent vaginal bleeding or discharge)

Unlikely: 1. . 2. . 3. . 4. . 5. . 6. .7: likely

1. Causing pain when doing a VE when a patient has abnormal vaginal bleeding is

Extremely undesirable: -3. .-2. . -1. . +1. . +2. . +3: extremely desirable

1. If I do a VE I will know the origin/source of the bleeding when a patient has abnormal vaginal bleeding.

Unlikely: 1. . 2. . 3. . 4. . 5. . 6. .7: likely

1. The history of presenting illness and previous treatments are necessary to make a decision to do a VE (patient with recurrent vaginal bleeding or discharge)

Unlikely: 1. . 2. . 3. . 4. . 5. . 6. .7: likely

**Case scenarios (Just your opinion – no correct or wrong answers.)**

1.A 14yr old girl comes to the h/centre complaining of vaginal bleeding and abdominal pains for a week. She is accompanied by her mother who reports she lives with her aunt during the week as her school is nearer there. Menarche was at 12 years.

Decision: Do a pelvic examination?

| Yes |  |
| --- | --- |
| No |  |

2.A 65 yr. old woman comes complaining of vaginal spotting after sexual intercourse. The spouse died 10 years ago but she has had a new partner the last 5 yrs. She has used KY lubricating gel but it still occurs. She Googled and wants to know if she can use a hormonal preparation. You have some free samples in the office and she has no other complains.

Decision: Would you do a pelvic exam?

| Yes |  |
| --- | --- |
| No |  |

3.The first patient this morning comes in with lower back pains for 4months. She is 39 yrs. and is on Depo Provera for contraception. The menses are usually irregular. She has been treated severally for a discharge which is blood tinged with antibiotics. She finished the last dose 6 weeks ago and she feels she needs a new prescription.

Decision: Do a pelvic examination?

| Yes |  |
| --- | --- |
| No |  |

4.Mrs comes for consultation complaining of left swollen leg and pain on walking. She’s 42 yrs. and has 4 children her last delivery was 6 yrs. ago. She has never married. Her method of contraception is combined pills which she started 5 months ago because of bleeding from implants. Initially she thought the pills were helping but of late a drug, Sylate (she was told to help blood clotting) was added to control the irregular bleeding. She also complains of occasional constipation and back pains. Is a pelvic examination pelvic exam necessary?

| Yes |  |
| --- | --- |
| No |  |

5.Mrs Z has heard about the cervical cancer vaccine and wants to know how/where to go for it. She is 30 yrs. para 3+0 and had a coil inserted 3 years ago. She has occasional blood-tinged discharge which she was told was because of the coil. This has become worse and she is getting scared of cancer since her grandmother died of breast cancer.

Decision: Do a pelvic examination?

| Yes |  |
| --- | --- |
| No |  |

6.Sara is a neighbour, 32yrs with 3 children. She is married and uses condoms for contraception because she was told her b/pressure was high in the last pregnancy.

She has confided in you severally on her irregular bleeding. She has come to report the medications you gave her 2months ago worked. But now she thinks she is pregnant but she is not ready for another pregnancy. She has lower abdominal pains.

Is a pelvic examination pelvic exam necessary?

| Yes |  |
| --- | --- |
| No |  |

7.Mary arrives at the clinic c/o lower abdominal pains (LAPs), and bleeding from the vagina. Her last normal menses were 3 years ago. She has had the LAPs for many months and she associates it with her workload. The bleeding is on and off but the last episode 5 months it lasted two weeks and left her weak. She feels much better has come for her monthly prescription of hematinics and analgesics.

Do a check-up, including pelvic exam?

| Yes |  |
| --- | --- |
| No |  |

8.Zippy was last seen at the clinic 5 yrs. ago for infertility but was told the cervix was normal. She is 38 years and her only child is 23 years. She has missed her menses the last 2 months. She feels she is pregnant and might lose her baby since she keeps spotting on and off.

Decision: Do a pelvic examination?

| Yes |  |
| --- | --- |
| No |  |

**9.**Jennifer has come several times complaining of a burning sensation on passing urine. She has been treated for infections severally. Last time she was treated together with the spouse. The partner refuses to use a condom. She says she has come for her usual medications as the blood-stained discharge has recurred again. She does not want to queue. Always she takes the same drugs and gets ‘cured’.

She can do **without** a pelvic exam.

| Yes |  |
| --- | --- |
| No |  |

**10.**65yr old Ms. Nancy noticed a mass in the lower abdomen 1yr ago. She also had lower abdominal pains which have become worse. She has been using traditional herbs which stopped the bleeding. During her last visit she was given something for the pains which are bearable now. She has been referred because she has difficulty controlling her urine and the urinalysis has shown blood.

An ultrasound in more important, a pelvic exam **can be deferred**?

| Yes |  |
| --- | --- |
| No |  |
